# Not Just Painless Bleeding: Meckel's Diverticulum as a Cause of Small Bowel Obstruction in Children—Two Cases and a Review of the Literature

**DOI:** 10.1155/2015/938346

**Published:** 2015-12-16

**Authors:** Khalida Itriyeva, Matthew Harris, Joshua Rocker, Robert Gochman

**Affiliations:** ^1^Department of Pediatrics, Cohen Children's Medical Center of New York, 269-01 76th Avenue, New Hyde Park, NY 11040, USA; ^2^Pediatric Emergency Medicine, Cohen Children's Medical Center of New York, 269-01 76th Avenue, New Hyde Park, NY 11040, USA

## Abstract

Physicians are educated with the classical teaching that symptomatic patients with Meckel's diverticulum (MD) most often present with painless rectal bleeding. However, a review of the literature reveals that young patients with MD will most commonly present with signs of intestinal obstruction, an etiology not frequency considered in patients presenting to the emergency department with obstruction. We present two cases of intestinal obstruction diagnosed in our emergency department, with Meckel's diverticulum being the etiology.

## 1. Case  1

An 18-month-old male with a past medical history significant for constipation and reflux presented to our pediatric emergency department with an approximately 5-hour history of lethargy, intermittent crying, and abdominal pain. The parents denied fever, vomiting, or abdominal distension but reported a decrease in oral intake and urine output. He had not stooled that day and had no prior surgical history.

Upon arrival to the emergency department (ED), the patient was afebrile with a rectal temperature of 98.2°F, heart rate of 129 bpm, blood pressure of 115/71, respiratory rate of 28, and oxygen saturation of 100% on room air. On physical exam, he was notably lethargic and pale, with sunken eyes and dry mucus membranes. He had periods of wakefulness, during which he appeared uncomfortable. He was intermittently tachycardic. The abdominal exam was notable for decreased bowel sounds and moderate distension with diffuse tenderness to palpation.

Intravenous access was obtained and the patient was given a 20 mL/kg normal saline bolus. Laboratory studies drawn were notable for a leukocytosis of 26,000 with a neutrophilic predominance and thrombocytosis. The chemistry obtained was grossly normal.

Intussusception was high on our differential diagnosis. Multiple-view abdominal plain films and an ultrasound of the abdomen were requested.

**Figure 1:**
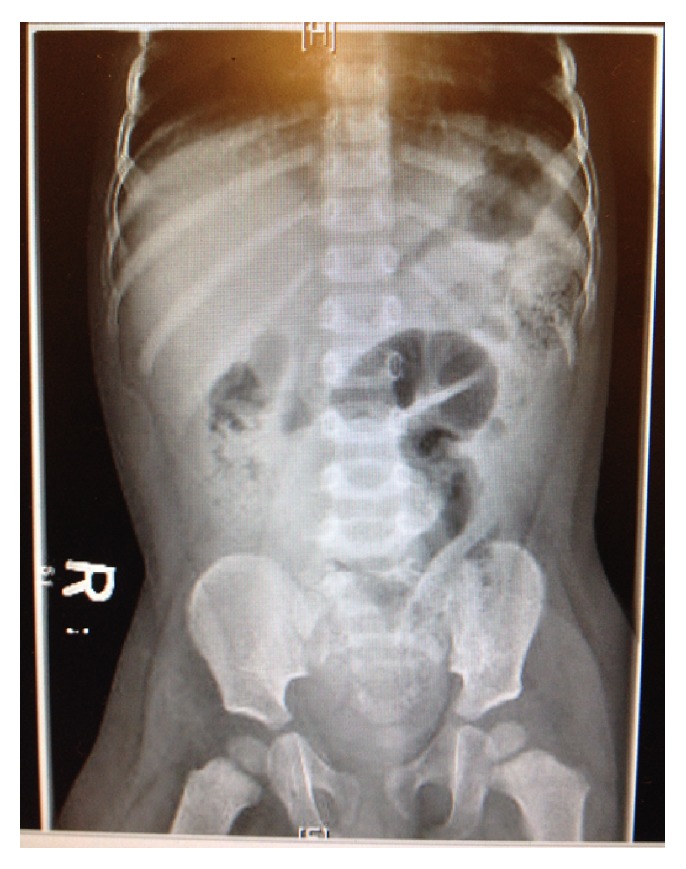
Supine abdominal plain film.

The supine abdominal plain film (Figure 1) revealed a moderately dilated loop of bowel in the mid-abdomen, a paucity of air in the right colon, and a large amount of stool. The abdominal ultrasound revealed marked bowel wall thickening in the right hemiabdomen with free fluid present both in the abdomen and in the pelvis. There was no evidence of appendicitis or intussusception.

The patient received a second 20 mL/kg saline bolus for persistent tachycardia and a pediatric fleet enema which did not produce significant stool. The patient developed worsening abdominal tenderness and bilious emesis and appeared obtunded, increasing our concern for an acute obstructive process. Intravenous piperacillin-tazobactam was empirically administered to the patient, given that a third 20 mL/kg normal saline bolus was administered, and a nasogastric tube was placed. A pediatric surgery consult was obtained and a noncontrast CT scan of the abdomen and pelvis was performed. The CT scan revealed a distal small bowel obstruction with evidence of ischemia and significant ascites.

**Figure 2:**
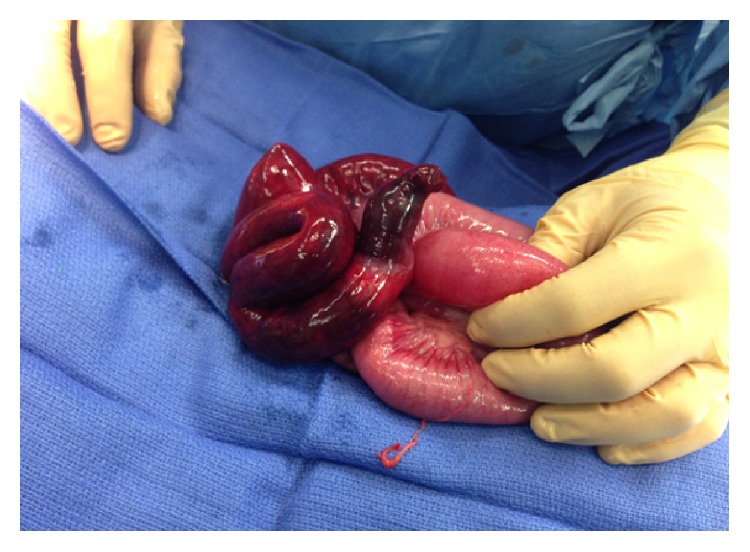
Intraoperative photograph showing portion of ischemic bowel.

A diagnostic laparoscopy and subsequent exploratory laparotomy revealed a congenital band extending from Meckel's diverticulum to the root of the mesentery, with thickened loops of dilated and ischemic bowel strangulated within this space (Figure 2). Significant ascites was also noted. A resection of the terminal ileum and cecum was performed, with subsequent primary ileocolic anastomosis. Forty-five cm of the distal ileum was found to be ischemic and subsequently resected. The postoperative period was unremarkable and the patient made a full recovery.

## 2. Case  2

**Figure 3:**
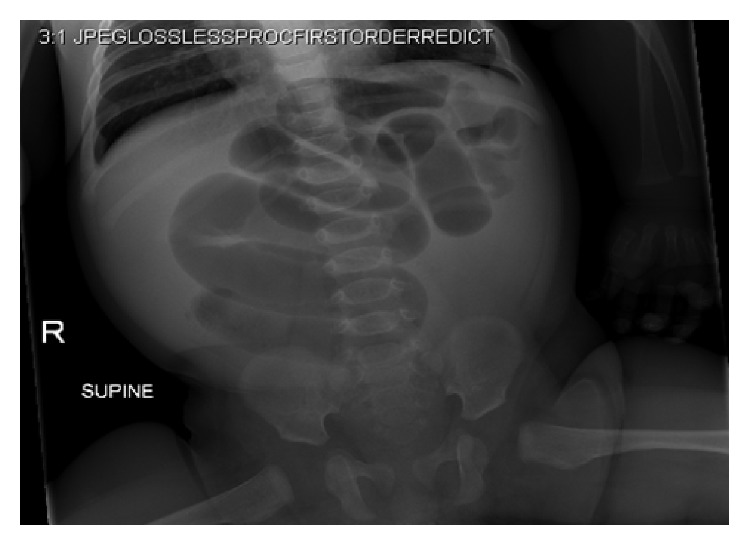
Supine abdominal plain film.

A 3-month-old full-term male with no prior medical history was referred to our pediatric emergency department after presenting to an outside institution with voluminous emesis and dehydration. Abdominal plain films were suspicious for malrotation (Figure 3). There had been no recent fever or URI symptoms, nor any diarrhea or rash.

On presentation to our pediatric ED, the patient was afebrile, with a pulse of 123 bpm, blood pressure 103/57 mmHg, respiratory rate of 26, and oxygen saturation of 100% on room air. On physical exam, the patient was alert, active, and playful and in no acute distress. He had dry lips, but his skin was warm and with brisk capillary refill. His abdomen was soft, nontender, and nondistended and without hepatosplenomegaly. He had a normal testicular and inguinal exam.

An abdominal ultrasound revealed multiple air filled loops of bowel within the mid-abdomen, with no evidence of intussusception. An upper GI series and barium enema were subsequently performed, revealing an abnormal position of the duodenum without the expected course of contrast to the left upper quadrant, suggesting the possibility of malrotation without volvulus.

Pediatric surgery was consulted, and the patient was taken to the operating room for diagnostic laparoscopy. Surgical evaluation revealed Meckel's diverticulum with a congenital band, causing an extra-luminal obstruction of the adjacent bowel—the cause of his vomiting. Ironically, the patient did also have a malrotation, without volvulus or obstruction from the Ladd's bands that were present; however this abnormal anatomy was not the cause of the patient's symptoms.

The congenital band and Meckel's diverticulum were resected to resolve the obstruction, without loss of bowel. Ladd's procedure was also performed to correct the malrotation. Final pathology report revealed Meckel's diverticulum without perforation lined by small intestinal and gastric antral mucosa showing active inflammation and reactive changes, with a nodule of pancreatic tissue in its wall.

## 3. Discussion

The differential diagnosis for a child with vomiting is broad and ranges from relatively benign conditions such as gastroenteritis to life-threatening causes such as volvulus. While the physical exam, laboratory studies, and imaging can often elucidate the cause of vomiting, it is sometimes incumbent upon the pediatric emergency medicine physician to recognize a surgical abdomen that requires an intraoperative evaluation for formal diagnosis and intervention.

Meckel's diverticulum (MD) is the most common congenital anomaly of the gastrointestinal tract, with an incidence of 2–4% in the general population [1]. Meckel's diverticulum is a persistence of the vestigial vitelline duct and is comprised of the three layers of the intestinal wall: mucosa, submucosa, and muscularis. While it may contain jejunal, duodenal, and even pancreatic tissue, it classically contains heterotopic gastric tissue. Most patients with MD are asymptomatic. Children are more likely to be symptomatic at presentation than adults. Twenty-five to fifty percent of all symptomatic Meckel's patients present before the age of 10. The incidence in males is twice that of females [2]. MD is most commonly found in the distal ileum, within 2 feet of the ileocecal valve, and, on gross resection, is approximately two inches in length. (The oft quoted “rule of 2's” is defined as follows: 2% incidence, 2 feet from the ileocecal junction, 2 inches in length, and 2 : 1 incidence in males over females.)

Most children with Meckel's diverticulum are asymptomatic. Complications of Meckel's diverticulum are seen more frequently in those who present at a younger age and in male patients [3, 4]. The classical description of painless bleeding is more commonly seen in adult patients, whereas in children, especially those younger than four years of age, the presentation is more likely to be that of an obstruction, as was the case with the patients in our case series. One large study of over 1400 patients, including 58 pediatric patients, found that obstruction was the most common presenting sign (40%) in children [3]. In their 2006 review of the literature, Sagar and colleagues found this number to be closer to 50% [1].

The etiologies of obstruction in children with symptomatic Meckel's diverticula include intussusception, volvulus, Littre's hernia, omphalomesenteric band, and diverticulitis [1–4]. Pediatric patients presenting with obstruction may exhibit irritability, paroxysmal abdominal pain, abdominal distension, nausea, vomiting, and anorexia.

Classically, one should suspect Meckel's diverticulum in children who present with painless lower gastrointestinal bleeding without evidence of infectious gastroenteritis or inflammatory bowel disease. MD may also be suspected in children with recurrent intussusception, as it may serve as a lead point. MD is also in the differential for right lower quadrant pain, especially in those patients with a previous appendectomy [2].

There are classically three diagnostic modalities in the evaluation of a patient with suspected Meckel's diverticulum. Meckel's scan is a nuclear medicine study that uses technetium 99m pertechnetate, which detects gastric mucosa, to identify ectopic gastric mucosa [2, 5]. Its sensitivity is much higher in pediatrics (85–90%) than in the adult population (60%) [5]. Mesenteric angiography may be helpful in identifying MD when a patient presents with active gastrointestinal bleeding. With this imaging modality, an anomalous superior mesenteric artery branch feeding the diverticulum may be visualized [2, 6]. Active contrast extravasation may also be seen in patients with persistent bleeding [2]. Finally, abdominal exploration in the operating room may lead to a finding of Meckel's diverticulum.

Our patients presented with evolving obstructive processes. To be clear, in the setting of a suspected obstructive process, the aforementioned imaging modalities are superfluous. Upright and decubitus abdominal radiographs to assess the patient for the presence of air-fluid levels, with computer aided tomography of the abdomen and pelvis with oral and intravenous contrast, may be necessary to identify the obstruction.

For the pediatric patient who presents with evidence of an obstructive process, there are a number of important aspects of management that should be addressed early. Intravenous access should be obtained immediately, and aggressive fluid resuscitation should be considered. A nasogastric tube should be placed for continuous suction and the patient made NPO. Any electrolyte abnormalities should be corrected, and a surgical consult should be obtained promptly. One can consider the empiric use of antibiotics with broad Gram-negative and anaerobic coverage, and some studies suggest the use of proton pump inhibitors [2]. Perhaps most importantly, urgent consultation with a pediatric surgeon can expedite both diagnosis and management.

The first patient in our series became progressively lethargic and obtunded. This is likely due to the release of inflammatory mediators as part of a SIRS response to strangulated tissue, as was evident by his tachycardia, tachypnea, and leukocytosis [7]. Previous studies have established that patients with a systemic inflammatory response in the setting of a suspected small bowel obstruction are more likely to have strangulated bowel [8].

In addition to Meckel's diverticulum, the second patient in our series was noted to have malrotation. The association between malrotation and other congenital gastrointestinal anomalies has been well reported. In one study, Meckel's diverticulum was the 2nd most common anomaly found with malrotation, behind duodenal atresia [9].

Both of our patients required resection of MD and associated bands to relieve their respective obstructive processes.

Meckel's diverticulum is occasionally found incidentally during abdominal surgery. Resection of incidental MD is generally not recommended, although this is an area of controversy and there may be exceptions [2, 3, 10]. One study suggested resection of asymptomatic Meckel's diverticula when they fulfilled one or more of the following four criteria: patient age younger than 50 years, male sex, diverticulum length greater than 2 cm, and ectopic or abnormal features within the diverticulum, as these were all associated with symptomatic diverticula in the study [3]. Resection is also recommended in all children under the age of 8 years, as they are more at risk of complications of MD [11]. In those patients who meet operative requirements, surgeons may choose either a simple diverticulectomy or small bowel resection with primary anastomosis [2]. Surgical complications and perioperative morbidity and mortality are very low [2, 10].

Younger children with Meckel's diverticulum are more likely to be symptomatic than older children. While the classical association of MD with painless rectal bleeding is still taught, obstruction is the more common presenting symptom in pediatric patients. Our case series identified two patients with different clinical presentations of obstruction, both of whom were found to have complications of MD. Small bowel obstruction is relatively uncommon in pediatrics, and our cases highlight the importance of early recognition and management to minimize morbidity and mortality. We strongly recommend early, urgent consultation with a pediatric surgeon in setting of suspected small bowel obstruction.
